# A grape (*Vitis vinifera* L.) pomace water extract modulates inflammatory and immune response in SW‐480 cells and isolated mouse colon

**DOI:** 10.1002/ptr.7581

**Published:** 2022-09-07

**Authors:** Lucia Recinella, Annalisa Chiavaroli, Serena Veschi, Alessandro Cama, Alessandra Acquaviva, Maria Loreta Libero, Sheila Leone, Simonetta Cristina Di Simone, Ester Pagano, Gokhan Zengin, Luigi Menghini, Luigi Brunetti, Angelo Antonio Izzo, Giustino Orlando, Claudio Ferrante

**Affiliations:** ^1^ Department of Pharmacy G. d'Annunzio University of Chieti‐Pescara Chieti Italy; ^2^ Veridia Italia Srl Pescara Italy; ^3^ Department of Pharmacy, School of Medicine University of Naples Federico II Naples Italy; ^4^ Physiology and Biochemistry Laboratory, Department of Biology, Science Faculty Selcuk University Konya Turkey

**Keywords:** catechin, colon cancer, grape pomace, inflammation, TRPM8, *Vitis vinifera*

## Abstract

Grape (*Vitis vinifera* L.) pomace is a residue derived from the winemaking process, which contains bioactive compounds displaying noteworthy health‐promoting properties. The aim of the present study was to investigate the phenolic composition and protective effects of a water extract of grape pomace (WEGP) in colorectal cancer cell line SW480 and in isolated mouse colon exposed to *Escherichia coli* lipopolysaccharide (LPS). The extract decreased SW‐480 cell viability, as well as vascular endothelial factor A (VEGFA), hypoxia‐induced factor 1α (HIF1α), and transient receptor potential M8 (TRPM8) LPS‐induced gene expression. Moreover, the extract inhibited mRNA levels of nuclear factor kB (NFkB), cyclooxygenase (COX)‐2, tumor necrosis factor (TNF)α, interleukin (IL)‐6, IL‐1β, IL‐10, inducible nitric oxide synthase (iNOS), and interferon (IFN)γ, in isolated colon. Conversely, WEGP increased the gene expression of antioxidant catalase (CAT) and superoxide dismutase (SOD), in the same model. The modulatory effects exerted by WEGP could be related, at least in part, to the phenolic composition, with particular regards to the catechin level. Docking calculations also predicted the interactions of catechin toward TRPM8 receptor, deeply involved in colon cancer; thus further suggesting the grape pomace as a valuable source of bioactive extracts and phytochemicals with protective effects in the colon.

## 1 INTRODUCTION

Grape (*Vitis vinifera* L., Vitaceae) is widely cultivated throughout the world (Afzalzadeh, Ahangarpour, Amirzargar, Varnamkhasti, & Ganjalidarani, 2015), with an annual production ranging from 75 to 85 million tons (Bigard, Romieu, Sire, & Torregrosa, 2020). In traditional medicine, grape has long been used to manage various health conditions, including gastroenteritis (Afzalzadeh et al., 2015). Interestingly, grape has been suggested as one of the most effective plants against colon cancer (Aiello et al., 2019). In particular, grape pomace is one of the main solid by‐products deriving from the winemaking process (Spinei & Oroian, 2021), which contains seeds, skin residues, and stems (Ruggieri et al., 2009). The composition of grape pomace was shown to be influenced by environmental factors, grape variety, as well as technology used for the winemaking process (Schieber, Stintzing, & Carle, 2001). Grape pomace contains neutral polysaccharides (30%), pectic substances (20%), insoluble proanthocyanidins (15%), and phenolic compounds, which mainly include resveratrol, anthocyanins, flavones, and tannins (Spinei & Oroian, 2021). In this context, a wide body of evidence hinted some biological activities exerted by grape polyphenols, including antioxidant, cardioprotective, anticancer, antiinflammatory, antiaging, and antimicrobial properties (Peixoto et al., 2018). In addition, the health‐promoting effects induced by the phenolic compounds of red wine are well known (Boussetta, Vorobiev, Le, Cordin‐Falcimaigne, & Lanoisellé, 2012; Renaud & De Lorgeril, 1992). Grape seed extract and its constituents were found to exert protective effects on TNBSS‐ or DSS‐induced ulcerative colitis in rodents (Cheah et al., 2013; Li, Cai, Qin, & Wu, 2008). Moreover, dietary grape seed extract supplementation has been previously considered to ameliorate inflammatory bowel disease symptoms in interleukin (IL)‐10 knockout mice (Wang et al., 2013), as well as to exert preventive effects against colorectal carcinogenesis (Tian et al., 2020). Considering these findings, in the present study, we aimed to evaluate the effects of a water extract of grape pomace (GPWE) in colorectal cancer cell line SW480 and in an ex vivo experimental model of colon inflammation constituted by isolated mouse colon challenged with *Escherichia coli* lipopolysaccharide (LPS) (Menghini et al., 2016). The grape pomace was the by‐product of the vintage (year 2020) of a DOC red wine, the Montepulciano d'Abruzzo variety (Villamagna doc).

## 2 MATERIALS AND METHODS

### 2.1 Plant material and extraction

Pomace was obtained from the grapes kindly provided by a local farm in the village of Villamagna (GPS coordinates: 42°19′47.42′′ N 14°14′12.8′′ E), province of Chieti (Italy). The authentication of the plant material was done by Proff. Luigi Menghini and. The pomace represents the residual waste product obtained after the preliminary fermentation and the subsequent pressing. The resulting material appears as semisolid tablets that were manually disrupted and immediately lyophilized to remove the residual water. The dry pomace was stored in a sealed plastic bag under vacuum and kept in the dark at room temperature until extraction.

The plant material derives from the cultivation (vintage of 2020) of local‐native ecotypes of *Vitis vinifera* variety “Montepulciano” used to produce a high‐quality red wine with Denomination of Controlled Origin (Villamagna DOC). According to the procedural guidelines, no less than 95% of the production is represented by the variety Montepulciano cultivated in selected geographical areas in the Abruzzo Region (Central Italy), limited to the municipalities of Villamagna, Vacri, and Bucchianico, in the province of Chieti (Italy). The soil exposure is south‐east or south‐west, and cultivation altitude is between 30 m and 180 m above the sea level with a low productive yield (less than 120 quintals per hectare) strongly characterized by organoleptic properties. Fifteen dry pomace samples of at least 200 g were taken randomly and gathered together to ensure the representativeness of the sample. For extraction processes, the sample of plant material is no less than 10 g, again to ensure uniformity. Samples weighed using a Precisa XT220A balance (Micro Precision Calibration Inc., Grass Valley, CA, USA) were roughly homogenized with a T25 digital Ultra‐Turrax tissue homogenizer (IKA, Staufen, Germany) in 50 ml Falcon tubes with bidistilled water (30 s at 10,000 g) in order to obtain uniform plant material size and to optimize the extraction. The homogenate was subjected to ultrasound‐assisted extraction (UAE) in a Trans‐sonic T460 ultrasonic bath (Elma, Singen, Germany). The operative conditions for the extraction were optimized through response surface methodology (Chiavaroli et al., 2021). Specifically, The optimal conditions to obtain the higher yield of the most abundant metabolites include high solvent: plant ratio (12 v/w), corresponding to 12 ml/g.

### 2.2 Total phenolic compounds, scavenging/reducing properties, and enzyme inhibition properties

Total phenols, flavonoids, flavonols, antocyanins, and intrinsic scavenging/reducing properties of the extracts were determined through colorimetric assays (Uysal et al., 2017; Zengin et al., 2020). Additionally, extracts were assayed for evaluating enzyme inhibition effects toward tyrosinase, α‐amylase, α‐glucosidase, and cholinesterases. Detailed protocols were reported as supplementary material.

### 2.3 HPLC‐DAD‐MS determination of phenolic compounds

The HPLC apparatus consisted of two PU‐2080 PLUS chromatographic pumps, a DG‐2080‐54 line degasser, a mix‐2080‐32 mixer, UV, diode array (DAD) and detectors, a mass spectrometer (MS) detector (expression compact mass spectrometer, Advion, Ithaca, NY 14850, USA), an AS‐2057 PLUS autosampler, and a CO‐2060 PLUS column thermostat (all from Jasco, Tokyo, Japan). Integration was performed by ChromNAV2 Chromatography software. Before the injection in the HPLC apparatus, the grape pomace extract was centrifuged at 3500×*g* for 15 min, and the supernatant was diluted to 20 mg/ml. Grape pomace water extract was analyzed for phenol quantitative determination using a reversed‐phase HPLC‐DAD‐MS in gradient elution mode. The separation was conducted within the 36 min of the chromatographic run, starting from the following separation conditions: 95% water with 0.1% formic acid, 5% methanol with 0.1% formic acid, as already reported in the literature (Orlando et al., 2021). The separation was performed on an Infinity lab Poroshell 120‐SB reverse phase column (C18, 150 × 4.6 mm i.d., 2.7 μm, Agilent, Santa Clara, CA, USA). Column temperature was set at 30°C. Quantitative determination of phenolic compounds was performed via a DAD detector. The extract was also qualitatively analyzed with MS detector in negative ion mode. MS signal identification was realized through comparison with a standard solution and MS spectra present in the MassBank Europe database (https://massbank.eu/MassBank/).

### 2.4 Cell lines

Colorectal cancer cell line SW480 were cultured in RPMI1640 (Sigma, St. Louis, MO, USA) supplemented with 10% fetal bovine serum (FBS), 1% Pen/Strep and 1% L‐glutamine. Normal fibroblast cell line HFF‐1 was cultured in DMEM high glucose (4.5 g/L; Sigma, St. Louis, MO, USA), supplemented with 15% FBS, 1% Pen/Strep and 1% L‐glutamine. Both cell lines were maintained in a humidified incubator at 37°C, 5% CO_2_.

### 2.5 Cell viability assay

Cell viability was evaluated by MTT assay [3‐(4,5‐Dimethyl‐2‐thiazolyl)‐2,5‐diphenyl‐2H‐tetrazolium bromide] (Sigma, St. Louis, MO, USA) as previously described (Veschi et al., 2018). Briefly, SW480 and HFF‐1 cell lines were seeded in 96‐well plates (5 × 10^3^ cells/well) and they were pretreated with 10 μg/ml lipopolysaccharide (LPS) for 24 hours. Subsequently, both LPS‐pretreated and not LPS‐pretreated SW480 and HFF‐1 cells were subjected to GPWE at various concentrations (0.1–1,000 μg/ml), or with vehicle (control) for a further 48 hours. In a second set of experiments, both LPS‐pretreated and not LPS‐pretreated SW480 cells were treated with GPWE (1,000 μg/ml), WS12 (5 μM), catechin (500 ng/ml), GPWE (1,000 μg/ml) + WS12 (5 μM), and GPWE (1,000 μg/ml) + catechin (500 ng/ml). After treatment, the MTT solution was added to each well and incubated at 37°C for at least 3 hours, until purple formazan crystals were formed. In order to dissolve the precipitate, the culture medium was replaced with dimethyl sulfoxide (DMSO, Euroclone). Absorbance of each well was quantified at 540 and 690 nm, using a Synergy H1 microplate reader (BioTek Instruments Inc., Winooski, VT, USA).

### 2.6 Ex vivo studies

Adult C57/BL6 male mice (3‐month‐old, weight 20–25 g) were housed in Plexiglas cages (2–4 animals per cage; 55 cm × 33 cm × 19 cm) and maintained under standard laboratory conditions (21 ± 2°C; 55 ± 5% humidity) on a 14/10 h light/dark cycle, with ad libitum access to water and food. Housing conditions and experimentation procedures were strictly in agreement with the European Community ethical regulations (EU Directive no. 26/2014) on the care of animals for scientific research. In agreement with the recognized principles of “Replacement, Refinement and Reduction in Animals in Research,” colon specimens were obtained as residual material from vehicle‐treated mice randomized in our previous experiments, approved by local ethical committee (“G. d'Annunzio” University, Chieti, Italy) and Italian Health Ministry (Project no. F4738.N.5QP).

Isolated colon specimens were maintained in a humidified incubator with 5% CO_2_ at 37°C for 4 h (incubation period), in RPMI buffer with added bacterial LPS (10 μg/ml), as previously described (Recinella et al., 2020; Recinella et al., 2021). During the incubation period, the tissues were challenged with scalar concentrations of GPWE (1–100 μg/ml).

### 2.7 RNA extraction, reverse transcription and real‐time reverse transcription polymerase chain reaction (RT‐PCR)

Total RNA was extracted from both the SW480 cells and colon specimens using TRI reagent (Sigma‐Aldrich, St. Louis, MO, USA), according to the manufacturer's protocol, and reverse transcribed using High Capacity cDNA Reverse Transcription Kit (ThermoFischer Scientific, Waltman, Massachusetts, USA). Gene expression of iNOS, COX‐2, NF‐kB and TNF‐α (on colon specimens) as well as TRPM8, HIF‐1α, and VEGF‐A (on SW480 cells) were determined by quantitative real‐time PCR using TaqMan probe‐based chemistry, as previously described (Leone et al., 2017; Orlando et al., 2018). PCR primers and TaqMan probes were purchased from Thermo Fisher Scientific Inc. The Assays‐on‐Demand Gene Expression Products used for gene expression evaluations in the mouse colon specimens were: Mm00478374_m1 for COX‐2 gene, Mm00443258_m1 for TNF‐α gene, Mm00440502_m1 for iNOS gene, Mm00476361_m1 for NF‐kB gene, Mm01168134_m1 for interferon‐γ gene, Mm00446190_m1 for IL‐6 gene, Mm01288386_m1 for IL‐10 gene, Mm00434228_m1 for the IL‐1β gene, Mm00607939_s1 for β‐actin gene. The Assays‐on‐Demand Gene Expression Products used for gene expression evaluations in the SW480 cells were: Hs01066596_m1 for TRPM8 gene, Hs00153153_m1 for HIF‐1α, Hs00900055_m1 for VEGFA, Hs00180269_m1 for BAX gene, Hs00608023_m1 for BCL2 gene, Hs99999903_m1 for *β*‐actin gene. *β*‐actin was used as the housekeeping gene. The elaboration of data was conducted with the Sequence Detection System (SDS) software version 2.3 (ThermoFischer Scientific). Relative quantification of gene expression was performed by the comparative 2^−∆∆Ct^ method (Livak & Schmittgen, 2001).

### 2.8 In silico studies

Docking calculations were conducted through the Autodock Vina of PyRx 0.8 software, as previously described (Angelini et al., 2020). Crystal structures of target protein were derived from the Protein Data Bank (PDB) with PDB ID as follows: 6NR3 [transient receptor potential M8 (TRPM8)]. Discovery studio 2020 visualizer was employed to investigate the protein–ligand nonbonding interactions.

### 2.9 Statistical analysis

Statistical analyses were performed using GraphPad Prism version 5.01 software (San Diego, CA). Means ± *SEM* were determined for each experimental group and analyzed by one‐way analysis of variance (ANOVA), followed by Newman–Keuls comparison multiple test. Statistical significance was set at *p* < .05. The number of animals randomized for each experimental group was calculated on the basis of the “resource equation” *N* = (*E* + *T*)/*T* (10 ≤ *E* ≤ 20) (Charan & Kantharia, 2013).

## 3 RESULTS AND DISCUSSION

### 3.1 Characterization of the extract

**FIGURE 1:**
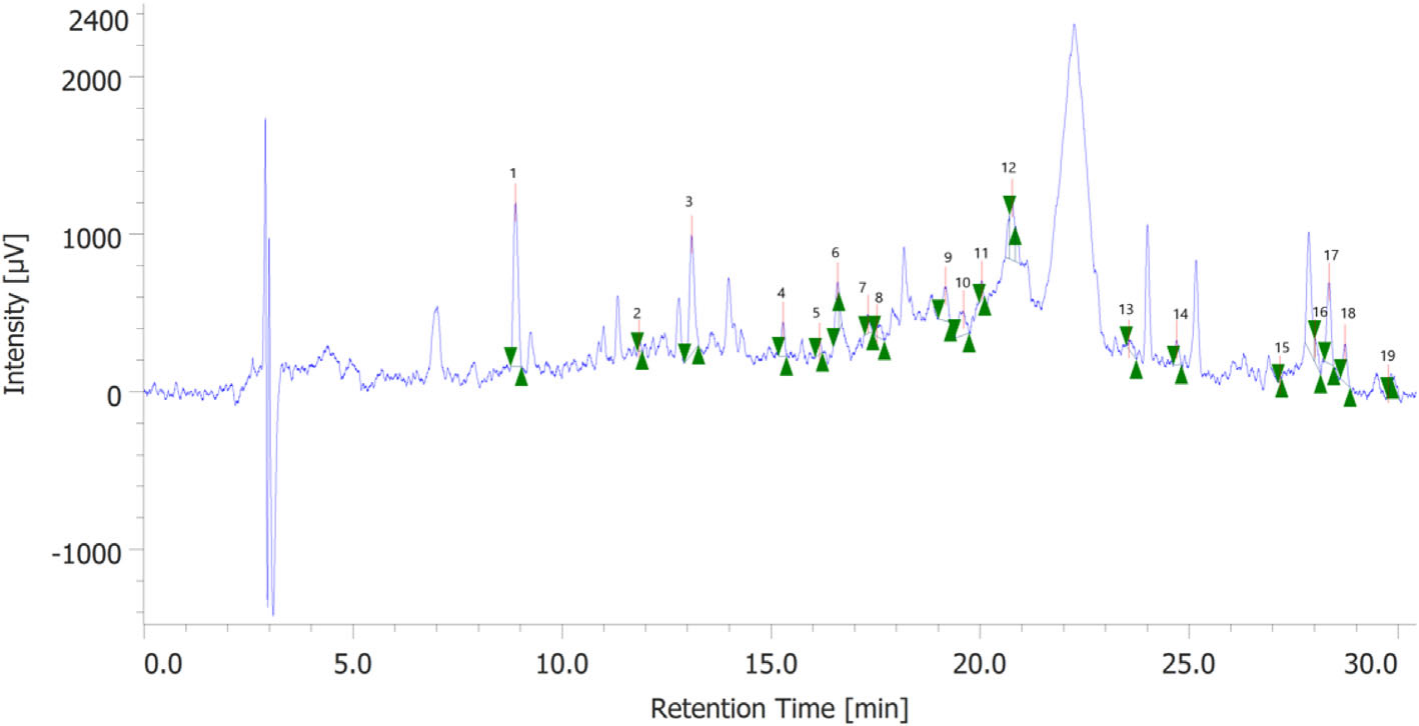
Chromatographic analysis of the water extract from the *Vitis vinifera* pomace. The chromatographic analysis confirmed the presence of 19 phytochemicals. The prominent compound was catechin (peak #4). The other main phytochemicals were gallic acid (peak #1), caftaric acid (peak #3), and epicatechin (peak #11)

**FIGURE 2:**
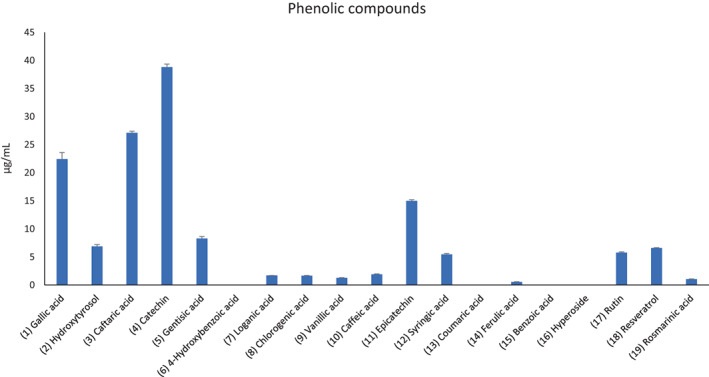
Phenolic profile of the grape pomace sample. Gallic acid, caftaric acid, and catechin were the main phytochemicals

In the present study, a water extract from grape pomace was examined to determine whether it could have beneficial effects on colon cancer and inflammation. This extract from plant material collected in the vintage of 2020 has been investigated for the content in phenolic compounds, with total phenols, total flavonoids, flavonols, and antocyanins assayed via colorimetric assays (Supplementary material: Table S1). Whereas 19 phytochemicals were identified through HPLC‐DAD‐MS, in comparison with pure standards (Table 1). The ultrasound‐assisted extraction conditions in water solvent were selected according to a recent study of ours (Chiavaroli et al., 2021), and catechin was found to be the prominent flavonoid (Figures 1 and 2). This is also consistent with the phytochemical profile of the water extract from the grape pomace collected during the 2019 vintage. The extract was also found effective in exerting scavenging/reducing and enzyme inhibition effects (Supplementary material: Table S2), which are consistent, albeit partially, with its content in phenolic compounds (Chatatikun et al., 2020; Meserole, 2002); thus supporting further pharmacological investigations for exploring health‐promoting effects, as described below.

**TABLE 1:** Wavelengths of quantification, mass to charge (*m*/*z*) ratios, and retention times related to the investigated phenolic compounds

#	Standard	*m/z*	Wavelengths (nm)	Retention time (min)
1	Gallic acid	169.1	254	8.883
2	Hydroxytyrosol	153.2	254	11.833
3	Caftaric acid	311.2	254	13.093
4	Catechin	289.3	254	15.287
5	Gentisic acid	153.1	254	16.160
6	4‐Hydroxybenzoic acid	137.1	254	16.587
7	Loganic acid	375.4	254	17.310
8	Chlorogenic acid	353.31	254	17.527
9	Vanillic acid	167.2	254	19.170
10	Caffeic acid	179.16	254	19.593
11	Epicatechin	289.3	254	20.037
12	Syringic acid	197.17	254	20.760
13	Coumaric acid	163.04	254	23.553
14	Ferulic acid	193.1	254	24.690
15	Benzoic acid	121.1	254	27.170
16	Hyperoside	463.4	254	28.003
17	Rutin	609.5	254	28.343
18	Resveratrol	227.2	254	28.720
19	Rosmarinic acid	359.3	254	29.760

### 3.2 Effects of the extract on colon cancer (SW480) cells

The extract was tested on colon cancer (SW480) cells for determining the effects on cell viability, in both basal and LPS‐induced inflammatory condition. In parallel, the extract was also tested in human fibroblast (HFF‐1) cells, which have been selected as non‐tumoral comparison cell line. Previous studies demonstrated the capability of catechins to induce apoptosis in SW480 cells (Cordero‐Herrera, Martín, Bravo, Goya, & Ramos, 2013; Kim, Mollah, & Kim, 2012), while the hydroalcoholic extract of grape pomace induced cytotoxic and antiproliferative effects, in human colon cancer HT‐29 cells (Pérez‐Ortiz et al., 2019). The dietary supplementation with grape pomace also demonstrated preventive effects against the onset of colon cancer in mice exposed to azoxymethane and dextran sulfate sodium (Tian et al., 2020).

**FIGURE 3:**
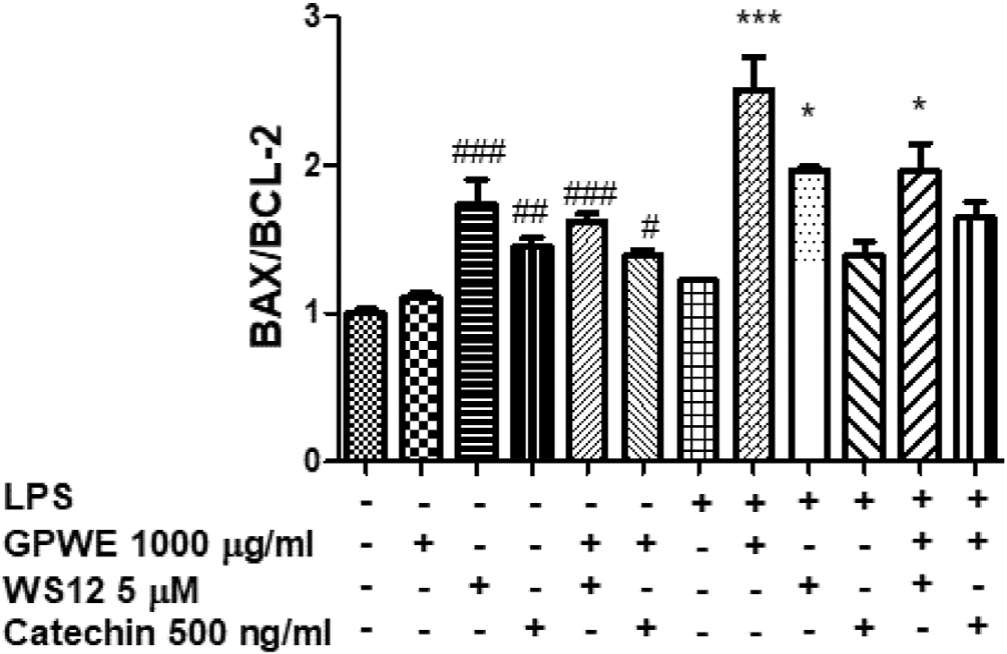
Effects of grape pomace water extract (GPWE) (1,000 μg/ml), WS12 (5 mM), catechin (500 ng/ml), GPWE (1,000 μg/ml) + WS12 (5 mM), and GPWE (1,000 μg/ml) + catechin (500 ng/ml) on BAX/BCL‐2 gene expression ratio, in both LPS‐pretreated and not LPS‐pretreated SW‐480 cells. Data are reported as means ± SEM. ANOVA, *p* < .001; ^#^ *p* < .05, ^###^ *p* < .001 versus Vehicle; ***p < .001, *p < .05 versus LPS

In the present study, the grape pomace extract (0.1–1,000 μg/ml) was able to induce a significant reduction of SW‐480 cell viability in both basal and LPS‐induced inflammatory conditions (Table 2); and the effects were relevant starting from the concentrations of 100 μg/ml, at which the cell viability was under the limits of biocompatibility (70% viability compared with the control group; *p* < .0001). By contrast, the HFF‐1 cell line viability was not modified by the extract (Table 2); thus, excluding any cytotoxic effect in non‐tumoral cells. In order to investigate the extract's cytotoxic effects, the gene expressions of BAX and BCL‐2 were measured as well, in SW‐480 cells. These two biomarkers are well known to play opposite effects on apoptosis, with BAX and BCL‐2 displaying pro‐apoptotic and anti‐apoptotic effects, respectively. Interestingly, BAX/BCL‐2 ratio represents a reliable index a proapoptotic activity (D'Angelo et al., 2017), and in the present study the BAX/BCL‐2 gene expression ratio (Li, Mu, Lin, Zhao, & Meng, 2021) was employed for investigating potential pro‐apoptotic effects induced by GPWE, in SW‐480 cells. The increase in BAX/BCL‐2 gene expression ratio (*p* < .001) following GPWE treatment (Figure 3) could underlie a possible influence of the extract on apoptosis pathway, in SW‐480 cells. The present findings are also consistent, though partially, with the inhibition of BCL‐2 gene expression induced by grape pomace and seed, in leukemic cells (León‐González, Jara‐Palacios, Abbas, Heredia, & Schini‐Kerth, 2017; Lin, Zhao, Huang, & Li, 2021). The gene expression of endothelia factor A (VEGFA) and the hypoxia‐induced factor 1α (HIF1α) was assessed as well, in SW‐480 cells. In this regard, a recent study pointed to the attenuation of LPS‐induced VEGFA level, as one of the mechanisms underlying the inhibition of SW480 cell migration induced by a purple rice extract (Panyathep, Punturee, & Chewonarin, 2021). Together with the HIF1α, VEGFA is a well‐known angiogenesis‐stimulating factor, and is considered a key mediator of the so‐called inflammatory to cancer transition (Chen et al., 2019). The inhibition of VEGFA and HIF1α gene expression (*p* < .0001) was also related to the cytotoxic effects induced by thalidomide in SW480 cells (Zhang & Luo, 2018). In the present study, the gene expression of both factors was downregulated by the extract, and the effect was more relevant when the cells were challenged with LPS (Table 3).

**TABLE 2:** MTT assay of LPS‐pretreated and not LPS‐pretreated SW‐480 and HFF‐1 cells exposed to grape pomace water extract (GPWE) (0.1–1,000 μg/ml)

	SW‐480 cells		HFF‐1 cells	
	Mean	*SEM*	Mean	*SEM*
Vehicle	100	3.45	100	3.29
GPWE 0.1 μg/ml	85.41	4.29	98.51	1.84
GPWE 1 μg/ml	85.49	5.52	96.91	3.36
GPWE 10 μg/ml	82.45*	4.94	107.30	2.58
GPWE 100 μg/ml	59.82***	2.40	106.5	3.23
GPWE 1000 μg/ml	60.44***	3.53	102.70	3.53
LPS	100	2.92	100	3.16
LPS + GPWE 0.1 μg/ml	108.50	5.95	114.50	4.57
LPS + GPWE 1 μg/ml	114.40	4.75	117	5.85
LPS + GPWE 10 μg/ml	96.74	4.33	116.40	4.71
LPS + GPWE 100 μg/ml	70.59^#^	2.62	116.10	3.35
LPS + GPWE 1000 μg/ml	72.36^#^	2.36	103.30	3.58

*Notes*: Data are reported as means ± *SEM*. ANOVA, *p* < .0001.

* *p* < .05.

*** *p* < .001 versus vehicle.

^#^ *p* < .001 versus LPS.

**TABLE 3:** Effects of grape pomace water extract (GPWE) (1,000 μg/ml), WS12 (5 mM), catechin (500 ng/ml), GPWE (1,000 μg/ml) + WS12 (5 mM), and GPWE (1,000 μg/ml) + catechin (500 ng/ml) on HIF‐1α and VEGFA gene expression, in both LPS‐pretreated and not LPS‐pretreated SW‐480 cells

	HIF‐1α		VEGFA	
	Mean	*SEM*	Mean	*SEM*
Vehicle	1.00***	0.00	1.00***	0.00
GPWE 1000 μg/ml	0.93	0.01	1.081	0.01
WS12 5 mM	0.99	0.03	0.80^#^	0.03
Catechin 500 ng/ml	0.76	0.05	0.95	0.02
GPWE 1000 μg/ml + WS12 5 μM	0.85	0.02	0.95	0.06
GPWE 1000 μg/ml + Catechin 500 ng/ml	0.86	0.02	1.02	0.04
LPS	1.47	0.04	1.67	0.05
LPS+ GPWE 1000 μg/ml	0.88***	0.06	0.80***	0.03
LPS + WS12 5 mM	1.17***	0.02	0.57***	0.05
LPS + Catechin 500 ng/ml	0.93***	0.02	0.88***	0.05
LPS + GPWE 1000 μg/ml + WS12	0.86***	0.02	0.69***	0.05
LPS + GPWE + Catechin	0.86***	0.01	0.88***	0.04

*Notes*: Data are reported as means ± *SEM*. ANOVA, *p* < .0001.

^#^ *p* < .05.

^##^ *p* < .01 versus Vehicle.

*** *p* < .001 versus LPS.

**FIGURE 4:**
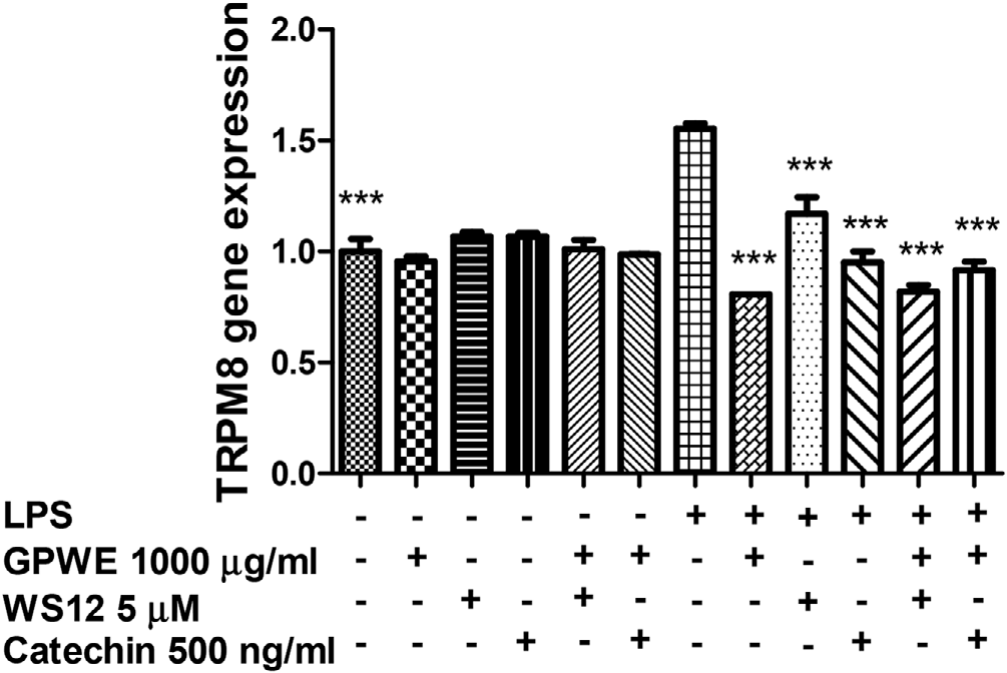
Effects of grape pomace water extract (GPWE) (1,000 μg/ml), WS12 (5 mM), catechin (500 ng/ml), GPWE (1,000 μg/ml) + WS12 (5 mM), and GPWE (1,000 μg/ml) + catechin (500 ng/ml) on TRPM8 gene expression, in both LPS‐pretreated and not LPS‐pretreated SW‐480 cells. Data are reported as means ± *SEM*. ANOVA, *p* < .0001; ***p < .001 versus LPS

In colon cancer, there is increasing evidence of the involvement of transient receptor potential (TRP) M8 (TRPM8), whereas its block has been related, albeit partially, to the inhibition of carcinogenesis, in mice (Borrelli et al., 2014; Liu, Li, & Xu, 2022). Additionally, in melanoma cells, the activation of TRPM8 was related to the inhibition of VEGFA‐induced transactivation of TRV1 (Walcher et al., 2018). In SW480 cells, the extract reduced the gene expression of TRPM8 (Figure 4; *p* < .0001), and this was mirrored by the corresponding inhibition of TRPM8 gene expression by catechin. Noteworthy, WS12 ((1R,2S,5R)‐2‐Isopropyl‐N‐(4‐methoxyphenyl)‐5‐methylcyclohexanecarboxamide), a selective TRPM8 agonist, determined an analogue inhibition of the receptor gene expression. Intriguingly, the inhibitory effects induced by the extract against the abovementioned genes, namely HIF1α, and VEGFA was comparable to that of catechin. In this regard, the concentration of catechin (500 ng/ml, 1.72 μM) chosen for the in vitro evaluation corresponds to the level of catechin in the extract, at the highest tested extract concentration (1,000 μg/ml); hence further suggesting the catechin as the potential phytochemical influencing the pattern of gene expression of the abovementioned factors. However, other phytochemicals, including antocyanins, could be involved in the cytotoxic effects induced by GPWE in SW480 cells (López de Las Hazas, Mosele, Macià, Ludwig, & Motilva, 2017; Sahpazidou et al., 2014). Furthermore, we cannot exclude that the observed cytotoxic effects on SW‐480 cells could derive upon the induction of apoptosis by polyphenolic compounds (Reddivari et al., 2016), as also partially suggested by our findings of increased BAX/BCL‐2 gene expression ratio.

### 3.3 In silico study

**FIGURE 5:**
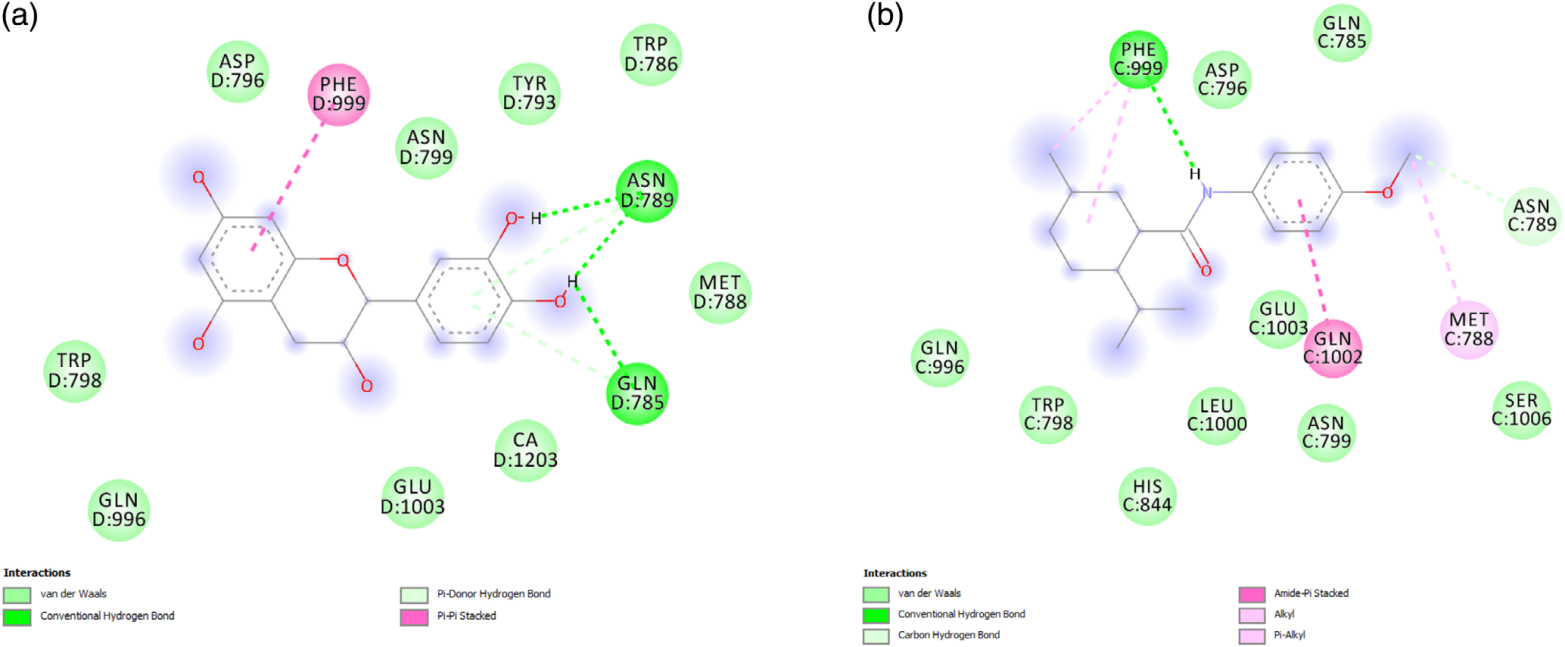
(a) Putative interactions between catechin and transient receptor potential M8 (TRPM8) (PDB: 6NR3). Free energy of binding (Δ*G*) and affinity (Ki) are −7.8 kcal/mol and 1.9 μM, respectively. (b) Putative interactions between WS12 and transient receptor potential M8 (TRPM8) (PDB: 6NR3). Free energy of binding (Δ*G*) and affinity (Ki) are −8.0 kcal/mol and 1.4 μM, respectively

Additionally, docking calculations showed that the putative affinity (1.95 μM) of catechin toward TRPM8 receptor (Figure 5a) is very close to both catechin level in the extract, and putative WS12 affinity (1.4 μM) towards TRPM8 receptor (Figure 5b). This suggests direct interactions between TRPM8 and catechin which could underlie, albeit partially, the pattern of receptor gene expression observed in SW‐480 cells. However, the cytotoxic effect induced by catechin was lower compared with the extract. This difference could be partly due to the presence of other phenolic compounds, including caftaric acid and gallic acid which could influence the cytotoxic effects of the extract (Arimoto‐Kobayashi et al., 2013).

### 3.4 Antiinflammatory effects in isolated colon tissue exposed to LPS

**FIGURE 6:**
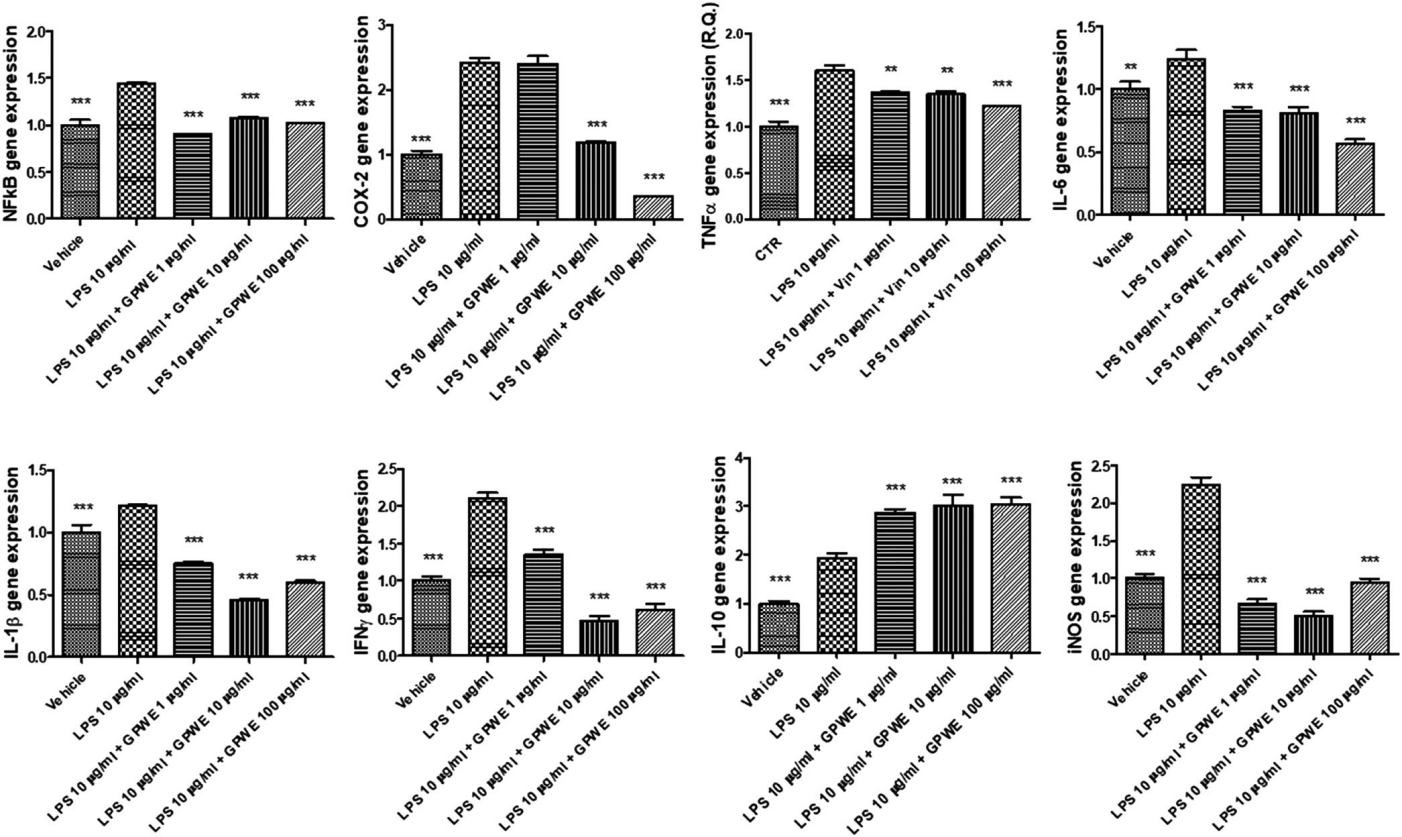
Effects of grape pomace water extract (GPWE) (1, 10, and 100 μg/ml) on NF‐kB, COX‐2, TNF‐α, IL‐6, IL‐1β, IFNγ, IL‐10, and iNOS gene expression, in mouse colon specimens. Data are reported as means ± SEM. ANOVA, *p* < .0001; **p < .01, ***p < .001 versus LPS

**FIGURE 7:**
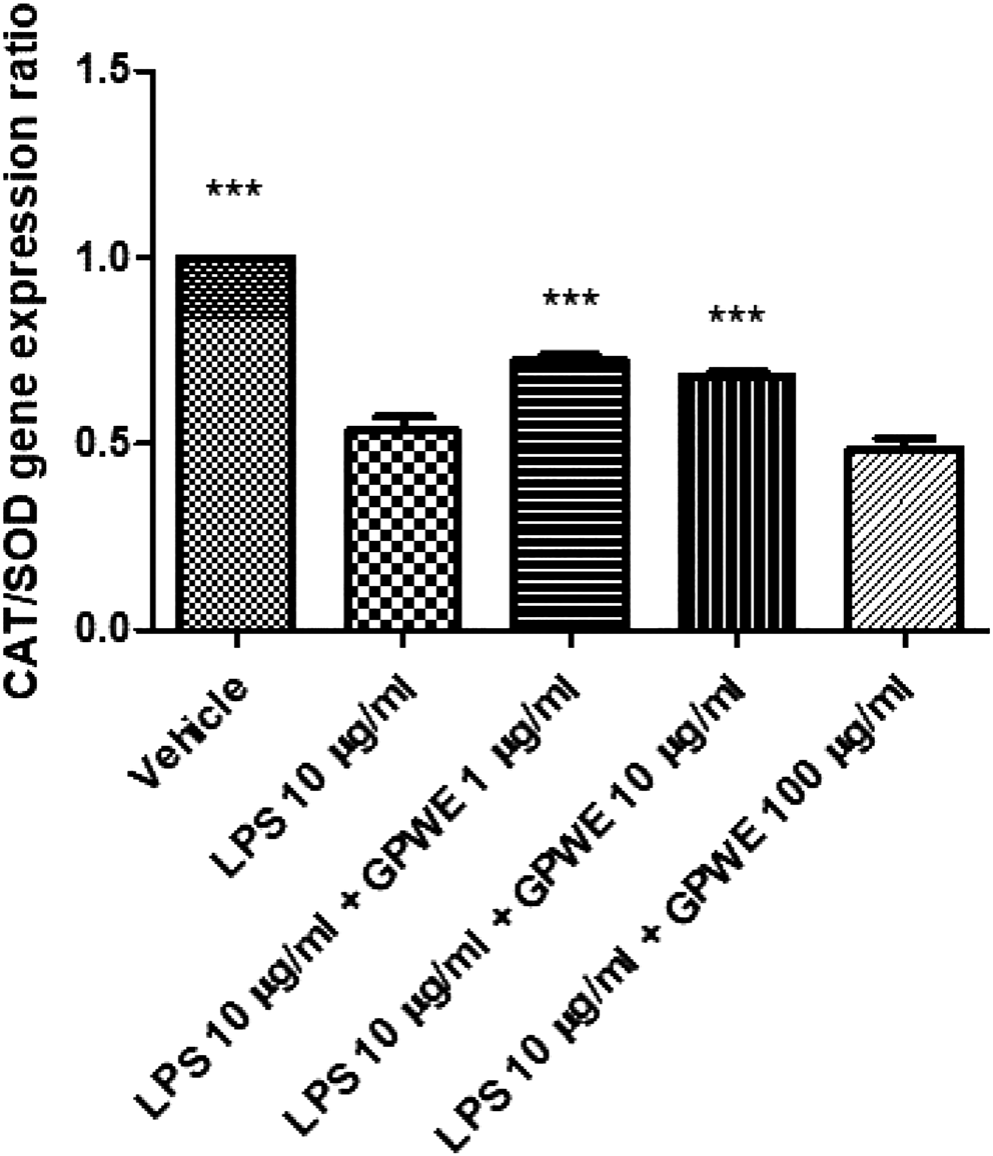
Effects of grape pomace water extract (GPWE) (1, 10, and 100 μg/ml) on CAT/SOD gene expression ratio, in mouse colon specimens. Data are reported as means ± SEM. ANOVA, *p* < .0001; ***p < .001 versus LPS

The extract was also tested in an ex vivo experimental model of colon inflammation, constituted by isolated mouse colon specimens exposed to LPS (Orlando et al., 2021). The extract was effective in reducing the LPS‐induced gene expression of different pro‐inflammatory biomarkers (*p* < .0001) involved in colon inflammation, among which nuclear factor kB (NFkB), cyclooxygenase (COX)‐2, tumor necrosis factor (TNF)α, interleukin (IL)‐6, IL‐1β, and interferon (IFN)γ (Figure 6) (Atreya, Atreya, & Neurath, 2008; Bouguen, Chevaux, & Peyrin‐Biroulet, 2011; Coccia et al., 2012) can be named. In parallel, the extract was effective in stimulating the gene expression of the anti‐inflammatory cytokine IL‐10 (Figure 6) (Moore, de Waal Malefyt, Coffman, & O'Garra, 2001). Besides, the gene expression of inducible nitric oxide synthase (iNOS) (Figure 6) , deeply involved in nitrosative stress, was inhibited. By contrast, the CAT/SOD gene expression ratio, index of antioxidant activity (Agostini et al., 2018), was augmented by GPWE extract (*p* < .0001), but only at the lowest tested concentrations (Figure 7). We cannot exclude that this could depend upon possible pro‐oxidant effects induced by phenolic compounds, in solution (Halliwell, Clement, Ramalingam, & Long, 2000).

Overall, the present findings agree with the present and recent findings of intrinsic scavenging/reducing and anti‐inflammatory effects by grape pomace extract (Chiavaroli et al., 2021). The pattern of phenolic composition, with particular regards to the content of catechin, could explain, albeit partially the modulatory effects on the abovementioned biomarkers of inflammation and oxidative stress (Fan, Sang, & Jiang, 2017).

## 4 CONCLUSIONS

The results of the study indicated the efficacy of the water extract from grape pomace in reverting the burden of inflammation and oxidative stress occurring in isolated colon specimens exposed to LPS. Whereas, the reduction of human colon cancer SW‐480 cell viability, and the modulation of pattern of gene expression of proteins involved in carcinogenesis, further support protective effects in the colon. The mechanism underlying these effects could involve more than one phytochemical. However, in SW480 cells the prominent phenolic compound, namely catechin, could be the main responsible of the inhibitory effects on VEGFA, HIF1α, and TRPM8 gene expression. Docking calculations also predicted the interactions of catechin towards TRPM8 receptor, deeply involved in colon cancer; therefore further suggesting the potential of grape pomace as a valuable source of bioactive extracts and phytochemicals with protective effects in the colon.

Considering also the large amount of grape pomace, about 20% of all processed plant material during wine‐making (Ageyeva et al., 2021), the present study strongly suggests the use of the pomace as a high‐quality by‐product. This could also lead to an overall improvement of the chain production, and this would be of particular importance for the local native‐ecotypes, in most cases showing high‐quality wines, such as the Villamagna DOC, but with low productivity.

## CONFLICT OF INTEREST

The authors declare no financial/commercial conflicts of interest.

## Supporting information


**Appendix S1** Supporting Information

## Data Availability

The data that support the findings of this study are available from the corresponding author upon reasonable request.
